# Polyunsaturated fatty acids metabolism, purine metabolism and inosine as potential independent diagnostic biomarkers for major depressive disorder in children and adolescents

**DOI:** 10.1038/s41380-018-0047-z

**Published:** 2018-04-20

**Authors:** Xinyu Zhou, Lanxiang Liu, Xinghui Lan, David Cohen, Yuqing Zhang, Arun V Ravindran, Shuai Yuan, Peng Zheng, David Coghill, Lining Yang, Sarah E Hetrick, Xiaofeng Jiang, Jean-Jacques Benoliel, Andrea Cipriani, Peng Xie

**Affiliations:** 1 Department of Psychiatry, The First Affiliated Hospital of Chongqing Medical University, Chongqing, China; 2 Institute of Neuroscience and the Collaborative Innovation Center for Brain Science, Chongqing Medical University, Chongqing, China; 3 Department of Psychiatry, Children’s Hospital of Chongqing Medical University, Chongqing, China; 4 Department of Child and Adolescent Psychiatry, Hôpital Pitié–Salpétrière, Institut des Systèmes Intelligents et Robotiques, Université Pierre et Marie Curie, Paris, France; 5 Department of Psychiatry, University of Toronto, Toronto, ON Canada; 6 Departments of Paediatrics and Psychiatry, University of Melbourne, Melbourne, Australia; 7 Centre for Youth Mental Health, University of Melbourne, Melbourne, Australia; 8 Department of Psychological Medicine, University of Auckland, Auckland, New Zealand; 9 UMRS 975, Pain Team, Site Pitie´-Salpeˆtrie’re, Universite´ Pierre et Marie Curie-Paris 6, Paris, 75013 France; 10 Department of Psychiatry, University of Oxford, Warneford Hospital, Oxford, UK; 11 Oxford Health NHS Foundation Trust, Oxford, UK

**Keywords:** Diagnostic markers, Predictive markers

## Abstract

Major depressive disorder (MDD) in children and adolescents is a recurrent and disabling condition globally but its pathophysiology remains poorly elucidated and there are limited effective treatments available. We performed metabolic profiling of plasma samples based on ultra-high-performance liquid chromatography equipped with quadrupole time-offlight mass spectrometry to explore the potential biomarkers of depression in children and adolescents with MDD. We identified several perturbed pathways, including fatty acid metabolism—particularly the polyunsaturated fatty acids metabolism, and purine metabolism—that were associated with MDD in these young patients. In addition, inosine was shown as a potential independent diagnostic biomarker for MDD, achieving an area under the ROC curve of 0.999 in discriminating drug-naive MDD patients and 0.866 in discriminating drug-treated MDD from healthy controls. Moreover, we found evidence for differences in the pathophysiology of MDD in children and adolescents to that of adult MDD, specifically with tryptophan metabolism. Through metabolomic analysis, we have identified links between a framework of metabolic perturbations and the pathophysiology and diagnostic biomarker of child and adolescent MDD.

## Introduction

Major depressive disorder (MDD) in children and adolescents is a major public health problem with an estimated point prevalence of between 2–3% in school-age children (6–12 years) and 3–9% in adolescents (13–18 years) [1, 2]. The diagnosis of MDD is often missed in young patients and even after recognition frequently under-treated [3]. Several factors may contribute to such under-recognition, but a key reason is the tendency to present “depressive equivalents” or undifferentiated depressive symptoms, e.g., school refusal, irritability, poor academic performance, etc. Another is the frequent presence of comorbidities, e.g., anxiety disorders, substance use [4], and attention deficit hyperactivity disorder [5]. It is well documented that children and adolescents with MDD frequently experience serious impairment in social and academic functioning, and are at significantly increased risk for suicidal ideation and behavior, which add to the significant burden of disease in this age group [6].

It has been noted that the profile of response following treatment of depression in children and adolescents is often very different from those of adults [7, 8]. One explanation proposed is that the pathophysiology of youth depression may not be the same to that of adult MDD. Several previous studies using proton magnetic resonance spectroscopy (^1^H-MRS) have confirmed the presence of neurochemical alternations in the brains of adolescents with depression that appears to have some degree of overlap those reported in adults [9–11]. However, other reports support the notion that different pathophysiological mechanisms mediating depression may differ in adults and adolescents [12, 13]. In particular the stage maturation of the key neuronal systems involved in affective regulation, including the serotonergic and adrenergic neurotransmitter systems [14]. As well, a peak in the prevalence of depression during adolescence add to the evidence for the influence of brain maturation and hormonal factors, increasing the vulnerability to depression in this age group [15].

Previously, we have used a metabolomics approach to document the molecular alterations in plasma [16, 17], urine [18], and peripheral blood mononuclear cells [19] in clinically depressed adult patients. As far as we are aware, similar reports in children and adolescents with depression are very limited. In this investigation, we attempted to identify metabolic changes unique to depression in children and adolescents with the ultimate aim of developing potential biomarkers that may aid in the diagnosis and effective treatment of depression in this age group. Finally, we also compared metabolomics data of children and adolescents with our previous metabolomics data of adult MDD [17] to document the common and unique metabolic changes in different aged cohorts.

## Experimental procedures

### Ethical statement

Written informed consent was obtained from the participants (if they were 18 years old) or their guardians (if aged 17 years or less). The experimental protocol was reviewed and approved by the Institutional Review Board of Children’s Hospital of Chongqing Medical University (No. 2016121).

### Participants

Patients aged from 6 to 18 years were recruited from the Children’s Hospital of Chongqing Medical University between November 2016 and May 2017 (Chongqing, China). The diagnosis of MDD was confirmed by trained psychiatrists fulfilling the criteria on Diagnostic and Statistical Manual of Mental Disorders—version 4 (DSM-IV). Two standardized and validated depression scales, i.e., Children’s Depression Rating Scale-Revised (CDRS-R) for children and younger adolescents (aged <15 years) [20] and Hamilton Depression Scale (17-Items) (HAMD-17) [21] for older adolescents (aged ≥15 years) were used to quantify the severity of depression and document change with treatment. Moderate to severely depressed patients (i.e., CDRS-R scores ≥40 or HAMD-17 scores ≥17), without any comorbid physical, neurological or psychiatric disorders and/or illicit drug use, were included in the study. We recruited both first-episode drug-naive MDD (DN-MDD) patients and drug-treated MDD (DT-MDD) patients. Healthy controls (HCs) with no previous neurological, DSM-IV Axis I/II, or medical illness were also recruited during the same study period. All HCs (*n* = 50) were recruited from routine medical examinations in two schools in Chongqing, China (one primary school in Yuzhong district and one middle school in Liangping district). If both students and parents agreed to participate in this study, the parents signed the information consent before recruitment. Information regarding sex, age, body mass index (BMI), depression symptoms severity, course of illness, and types of antidepressants was collected.

### Sample collection and preparation

The fasting blood was collected in 5 mL vacutainer tubes containing heparin lithium, then the samples were centrifuged for 15 min (1500 × *g*, 4 °C). Each aliquot (150 μL) of the plasma sample was stored at –80 °C until the ultra-high-performance liquid chromatography equipped with quadrupole time-offlight mass spectrometry (UPLC-Q-TOF/MS) analysis. The plasma samples were thawed at 4 °C and 100 μL aliquots were mixed with 400 μL of cold methanol/acetonitrile (1:1, v/v) to remove the protein. The mixture was centrifuged for 15 min (14,000 × *g*, 4 °C). The supernatant was dried in a vacuum centrifuge. For the UPLC-Q-TOF/MS analysis, the samples were re-dissolved in 100 μL acetonitrile/water (1:1, v/v) solvent. To monitor the stability and repeatability of instrument analysis, quality control (QC) samples were prepared by pooling 10 μL of each sample and these were analyzed together with the other samples. The QC samples were inserted regularly and analyzed in every eight samples.

### UPLC-Q-TOF/MS analysis

Metabolic profiling of plasma samples was performed on an Agilent 1290 Infinity LC system (Agilent Technologies, Santa-Clara, California, USA) coupled with an AB SCIEX Triple TOF 6600 System (AB SCIEX, Framingham, MA, USA). Chromatographic separation was implemented on ACQUITY HSS T3 1.8 µm (2.1 × 100 mm) columns for both positive and negative models. The column temperature was set at 25 °C. The mobile phases of 0.1% formic acid in water (A) and 0.1% formic acid in acetonitrile (B) were used in positive ionization mode, while 0.5 mM ammonium fluoride in water (C) and acetonitrile (D) were used in negative ionization mode. In the positive (negative) model, the elution gradient initially started with 1% B (D) for 1 min, linearly increased to 100% B (D) at 8 min, maintained for 2 min, and then returned to 1% B (D) for about 2 min of equilibrium. The delivery flow rate was 300 μL/min, and 2 μL aliquot of each sample was injected onto the column.

TOF/MS was performed on positive ion mode and negative ion mode. Electrospray ionization (ESI) source conditions on Triple TOF were set as following: ion source gas 1, 40 psi; ion source gas 2, 60 psi; curtain gas, 30 psi; source temperature, 650 °C; ionspray voltage floating, 5000 V (+) and −4500 V (−). Information-dependent acquisition (IDA), an artificial intelligence-based product ion scan mode, was used to detect and identify MS/MS spectra. The parameters were set as follows: declustering potential, 60 V (+) and −60 V (−); collision energy, 50 V (+) and −20 V (−); exclude isotopes within 4 Da, candidate ions to monitor per cycle: 10.

### Metabonomics data analysis

The raw UPLC-Q-TOF/MS data were converted to mzXML files using Proteo Wizard MSconventer tool and then processed using XCMS online software. The parameters in XCMS were set as follows: centwave settings for feature detection (Δm/z = 25 ppm, peakwidth = c (10, 60)); obiwarp settings for retention time correction (profStep = 1); and parameters including minfrac = 0.5, bw = 5 and mzwid = 0.025 for chromatogram alignment. After being normalized and integrated by using support vector regression, the processed data were uploaded into MetaboAnalyst software for further analysis (www.metaboanalyst.ca). Principal component analysis (PCA) and partial least square discriminant analysis (PLS-DA) were performed for both positive and negative models after log transformation and pareto scaling. The variable importance in the projection (VIP) value of each variable in the PLS-DA model was calculated to indicate its contribution to the classification. Metabolites with the VIP value >1 were further applied to Student’s *t*-test at univariate level to measure the significance of each metabolite, with results adjusted for multiple testing using the Benjamini–Hochberg procedure with the critical false discovery rate (FDR) set to 0.05. The heat plot of metabolites was gained by Multi Experiment Viewer (MeV) software 4.9.0 after unit variance scaling for each metabolite.

### Bioinformatics analysis and biomarker identification

Analyses were conducted in three separate settings.

- In setting 1, we compared the metabolic phenotypes of DT-MDD and DN-MDD, and analyzed the perturbed pathways in DN-MDD vs. HCs to explore the underlying pathophysiology of depression in children and adolescents. Ingenuity Pathway Analysis (IPA, http://www.ingenuity.com) software and MetaboAnalyst were used to perform biological function analysis.
- In setting 2, analysis of significant metabolites between DN-MDD and HCs were performed to identify potential diagnostic biomarkers. Two methods were used: (1) A stepwise binary logistic-regression model was used for the multivariate analysis [22]; (2) A receiver-operating characteristic curve (ROC) analysis was carried out to quantify the diagnostic performance of individual metabolites [23], and the value of area under the ROC curve (AUC) was calculated. Then, multiple regression analysis was performed to investigate whether the selected biomarker was affected by clinical characteristics in DN-MDD patients. The variables investigated included: sex, age, BMI, depression symptoms severity and course of illness.
- In setting 3, we compared the metabolic phenotypes of depression in adults with those in children and adolescents to reveal the common and unique underlying pathophysiology of depression in different age cohorts.

### Statistical analysis

Continuous variables were expressed as mean±standard deviation (SD) or median with interquartile range (IQR). Analyses of clinical characteristics were performed using Analysis of Variance (ANOVA) followed by post hoc Bonferroni, Mann–Whitney U or Chi-square tests, where appropriate. A *p*-value of less than 0.05 was considered to be statistically significant.

## Results

**Fig. 1:**
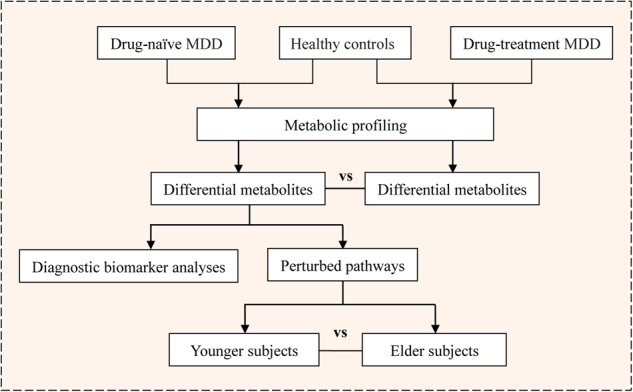
Flowchart of the study analyses in this work. MDD, major depressive disorder

A total of 134 children and adolescents, including 52 first-episode DN-MDD subjects (male/female: 27/25), 32 DT-MDD subjects (male/female: 15/17) and 50 HCs (male/female: 27/23) were recruited (Fig. 1). The clinical and demographic characteristics of the subjects are presented in Table 1. There were no significant differences in sex, age and BMI between the three groups of participants. The duration of illness and also the severity of depressive symptoms did not differ between the two MDD groups (DN-MDD and DT-MDD) on either the HAMD-17 (22.50 ± 3.63 vs. 21.36 ± 2.99) or the CDRS-R (46.56 ± 6.56 vs. 52.20 ± 7.45), respectively. The antidepressants presented to DT-MDD subjects were primarily selective serotonin reuptake inhibitors (SSRIs), i.e., escitalopram, fluoxetine, fluvoxamine, paroxetine, and sertraline. The median duration of the treatment in DT-MDD was 8 weeks (range 4–16).

**Table.1:** Clinical characteristics of the recruited major depressive disorder patients and healthy controls

Characteristics	Drug-naive MDD	Drug-treated MDD^d^	Healthy controls	*P*-value
Patients (*n*)	52	32	50	—
Male (*n*, %) ^a^	27 (51.9%)	15 (46.9%)	27 (54.0%)	0.818
Age (years)				
Range	9–18	11–18	7–18	—
Mean±SD ^b^	15.79±2.56	15.88±1.96	15.54±2.51	0.789
BMI (kg/m^2^)				
Mean±SD ^b^	19.61±2.27	19.95±2.47	19.76±1.71	0.582
Depression symptoms severity ^c^				
HAMD-17 (Mean±SD)	22.50±3.63	21.36±2.99	NA	0.231
CDRS-R (Mean±SD)	46.56±6.56	52.20±7.45	NA	0.080
Duration of illness (months) ^c^				
Median, IQR	8.55 (4.28–15.63)	12.80 (6.40–19.20)	NA	0.970
Duration of antidepressant treatment (weeks)				
Median, IQR	NA	3.79 (2.12–7.52)	NA	
Types of antidepressants (*n*, %)				
Fluvoxamine	NA	10 (31.3%)	NA	—
Fluoxetine	NA	5 (15.6%)	NA	—
Sertraline	NA	5 (15.6%)	NA	—
Escitalopram	NA	4 (12.5%)	NA	—
Paroxetine	NA	2 (6.3%)	NA	—
Others	NA	6 (18.7%)	NA	—

Continuous variables are expressed as Mean±Standard Deviation (SD) or Median with Interquartile Range (IQR)

*BMI* Body Mass Index, *CDRS-R* Children’s Depression Rating Scale-Revised, *HAMD-17* Hamilton Depression Scale (17-Items), *MDD* Major Depressive Disorder

^a^Analyzed by the Chi-square test

^b^Analyzed by the ANOVA followed by *post hoc* comparison of the groups using the Bonferroni test

^c^Analyzed by Mann-Whitney U test

^d^All drug-treated MDD patients were receiving their first antidepressant

### Metabonomic analysis of plasma obtained from MDD subjects and HCs

**Fig. 2:**
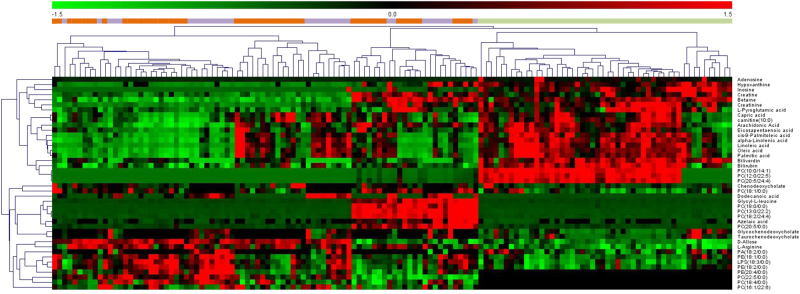
Heat plot of the differential metabolites in depression patients vs. healthy controls.  drug-naive MDD  drug-treated MDD  healthy controls. MDD major depressive disorder

For the initial untargeted metabolomics analysis, 134 plasma samples were examined. In the metabolic profiling, 5353 positive-mode features and 1680 negative-mode features were identified and applied for MetaboAnalyst analysis. After log transformation and pareto scaling of ion features, QC samples were clustered tightly on PCA score plots in both positive and negative modes (Figure S1), indicating satisfactory reproducibility. To identify differences in metabolic profiles between groups, PLS-DA score plots were performed for both positive and negative modes (Figure S2). There were remarkable separations for both DN-MDD vs. HCs and DT-MDD vs. HCs. The metabolites that differentiated between the groups were filtered using multivariate and univariate statistical significance criteria (VIP>1 and FDR<0.05). In total, 35 identified metabolites in DN-MDD vs. HCs, 36 in DT-MDD vs. HCs, and 6 in DT-MDD vs. DN-MDD showed significant between group differences. The detailed information on these metabolites is detailed in Table 2. The heat plot for the differential metabolites in MDD vs. HCs is presented in Fig. 2.

**Table 2:** Identified differential metabolites between the drug-naive MDD, drug-treatment MDD and health controls

Metabolites	DN-MDD vs HCs				DT-MDD vs HCs				DT-MDD vs DN-MDD				Pathways
	ESI^+/−^	FC	FDR	VIP	ESI^+/−^	FC	FDR	VIP	ESI^+/−^	FC	FDR	VIP	
L-Arginine	−	2.30	<0.001	2.00	−	2.23	<0.001	1.94	—	—	—	—	Amino acid metabolism
L-Pyroglutamic acid	+	0.52	<0.001	1.12	+	0.47	<0.001	1.20	—	—	—	—	Amino acid metabolism
carnitine(10:0)	+	0.61	0.0001	1.04	+	0.61	<0.001	1.02	—	—	—	—	Beta oxidation of fatty acids
Bilirubin	+	0.62	<0.001	1.02	+	0.59	<0.001	1.10	—	—	—	—	Bile acid metabolism
Biliverdin	±	0.45	<0.001	1.40	±	0.47	<0.001	1.34	—	—	—	—	Bile acid metabolism
Chenodeoxycholate	−	1.95	0.042	1.03	—	—	—	—	—	—	—	—	Bile acid metabolism
Taurochenodeoxycholate	—	—	—	—	−	1.86	0.037	1.66	—	—	—	—	Bile acid metabolism
Glycochenodeoxycholate	—	—	—	—	−	1.99	0.005	1.42	—	—	—	—	Bile acid metabolism
Creatine	+	0.65	0.015	1.18	+	0.56	0.006	1.28	—	—	—	—	Energy metabolism
Creatinine	+	0.37	<0.001	1.75	+	0.35	<0.001	1.82	—	—	—	—	Energy metabolism
alpha-Linolenic acid	-	0.45	<0.001	2.09	−	0.50	<0.001	1.90	—	—	—	—	Fatty acid metabolism
Arachidonic Acid	-	0.69	<0.001	1.06	−	0.57	<0.001	1.35	—	—	—	—	Fatty acid metabolism
Azelaic acid	-	16.33	0.002	2.24	−	18.12	0.003	2.66	—	—	—	—	Fatty acid metabolism
Capric acid	-	0.58	<0.001	1.49	−	0.61	0.005	1.26	—	—	—	—	Fatty acid metabolism
cis-9-Palmitoleic acid	-	0.42	<0.001	2.20	−	0.45	<0.001	2.02	—	—	—	—	Fatty acid metabolism
Dodecanoic acid	-	0.48	<0.001	2.01	−	0.50	0.002	2.03	—	—	—	—	Fatty acid metabolism
Eicosapentaenoic acid	-	0.61	0.001	1.34	−	0.45	<0.001	1.63	—	—	—	—	Fatty acid metabolism
Linoleic acid	-	0.41	<0.001	2.07	−	0.50	<0.001	1.85	—	—	—	—	Fatty acid metabolism
Oleic acid	-	0.50	<0.001	1.90	−	0.53	<0.001	1.72	—	—	—	—	Fatty acid metabolism
Palmitic acid	-	0.57	<0.001	1.65	−	0.61	<0.001	1.48	—	—	—	—	Fatty acid metabolism
LPS(18:3/0:0)	-	1.60	<0.001	1.24	−	1.67	<0.001	1.38	—	—	—	—	Lipid metabolism
PC(10:0/14:1)	+	0.03	<0.001	3.29	+	0.02	<0.001	3.41	—	—	—	—	Lipid metabolism
PC(12:0/22:5)	+	0.03	<0.001	3.29	+	0.02	<0.001	3.36	—	—	—	—	Lipid metabolism
PC(13:0/22:2)	+	12.07	<0.001	1.40	+	11.52	<0.001	1.40	—	—	—	—	Lipid metabolism
PC(18:0/0:0)	+	24.53	<0.001	1.27	+	16.61	<0.001	1.23	—	—	—	—	Lipid metabolism
PC(18:1/0:0)	−	1.50	<0.001	1.13		—	—	—	—	—	—	—	Lipid metabolism
PC(18:2/24:4)	+	3.14	<0.001	1.10	+	3.09	<0.001	1.09	—	—	—	—	Lipid metabolism
PC(20:5/0:0)	+	6.01	<0.001	1.40	+	6.69	0.003	1.31	—	—	—	—	Lipid metabolism
PC(20:5/24:4)	+	0.05	<0.001	3.07	+	0.03	<0.001	3.25	—	—	—	—	Lipid metabolism
PE(18:1/0:0)	−	1.54	<0.001	1.11	−	1.83	<0.001	1.42	—	—	—	—	Lipid metabolism
PE(18:2/0:0)	−	1.48	<0.001	1.00	−	1.61	<0.001	1.23	—	—	—	—	Lipid metabolism
PA(18:2/0:0)	—	—	—	—	−	1.20	0.003	1.72	—	—	—	—	Lipid metabolism
PC(16:1/22:6)	—	—	—	—	—	—	—	—	+	1.48	0.030	2.09	Lipid metabolism
PC(18:4/0:0)	—	—	—	—	—	—	—	—	+	1.15	0.045	1.10	Lipid metabolism
PC(22:5/0:0)	—	—	—	—	—	—	—	—	+	1.36	0.030	1.71	Lipid metabolism
PE(20:4/0:0)	—	—	—	—	—	—	—	—	+	1.31	0.043	1.98	Lipid metabolism
Adenosine	+	0.09	0.015	2.06	+	0.06	0.047	2.36	+	0.72	0.039	2.15	Purine metabolism
Inosine	±	0.02	<0.001	3.85	+	0.21	<0.001	3.41	+	10.83	0.030	3.02	Purine metabolism
Hypoxanthine	−	0.22	<0.001	3.56	−	0.52	0.006	2.91	—	—	—	—	Purine metabolism
Betaine	+	0.67	<0.001	1.16	+	0.68	<0.001	1.13	—	—	—	—	Other
D-Allose	−	1.77	<0.001	1.42	−	1.80	<0.001	1.52	—	—	—	—	Other
Glycyl-L-leucine	+	9.89	<0.001	1.39	+	15.53	<0.001	1.44	—	—	—	—	Other

*DN-MDD* drug-naive major depressive disorder, *DT-MDD* drug-treated major depressive disorder, *ESI* electrospray ionization, *FC* fold change, *FDR* false discovery rate, *HCs* healthy controls, *VIP* variable importance in the projection

### Bioinformatics analysis of children and adolescents DN-MDD

IPA and MetaboAnalyst were used to gain further understanding into the metabolic disturbances in DN-MDD vs. HCs. The significantly perturbed pathways are shown in Table 3. These indicate that there were differences in both the fatty acid and purine metabolism pathways in children and adolescent with DN-MDD compared with HCs. The molecular interaction network that was most altered by IPA was “Carbohydrate Metabolism, Energy Production, Small Molecule Biochemistry” with a score of 36 (Figure S3). We further investigated the significant alterations in fatty acid metabolism and purine metabolism. Compared with HCs, DN-MDD exhibited a decrease in fatty acids, including; capric acid, cis-9-palmitoleic acid, dodecanoic acid, oleic acid and palmitic acid, and the polyunsaturated fatty acids (PUFAs), including; eicosapentaenoic acid (EPA,ω-3) and arachidonic acid (AA,ω-6) which is generated from phospholipids through phospholipase A2 with lysophospholipids as a by-product. α-Linolenic acid (ALA,ω-3) and linoleic acid (LA,ω-6), which are the precursors of EPA and AA, respectively.

**Table 3:** The significantly altered pathways in major depressive disorder with children and adolescents and adults

Children and adolescents MDD		Adults MDD	
Pathways	*p*-value	Pathways	*p*-value
IPA analyses			
Adenine and Adenosine Salvage III	6.56E-07	tRNA Charging	2.80E-04
Purine Ribonucleosides Degradation to Ribose-1-phosphate	1.10E-06	FXR/RXR Activation	7.80E-04
Adenosine Nucleotides Degradation II	3.14E-06	S-adenosyl-L-methionine Biosynthesis	2.52E-03
Purine Nucleotides Degradation II (Aerobic)	7.40E-06	Methionine Salvage II (Mammalian)	2.84E-03
Glycine Degradation (Creatine Biosynthesis)	3.69E-05	Lipoate Biosynthesis and Incorporation II	3.15E-03
MetaboAnalyst analyses			
Fatty acid biosynthesis	9.03E-5	Glycerophospholipid metabolism	7.83E-4
Arginine and proline metabolism	3.93E-02		

*IPA* ingenuity pathway analysis, *MDD* major depressive disorder

In addition, adenosine, inosine, and hypoxanthine, the various products of adenosine triphosphate (ATP) degradation, were decreased, indicating a dysfunction of purine metabolism in young patients with DN-MDD. These pathways are illustrated in Figure S4.

### The clinical characteristics analysis for subgroups in DN-MDD and HCs groups

Regarding the PLS-DA score plots of DN-MDD verses HCs, significant intragroup separations were observed in both the DN-MDD and HCs groups. These were most obvious in the positive model. In order to understand these separations better, we compared the clinical characteristics between the subgroups (Table S1). Interestingly, the two separated subgroups in DN-MDD were significantly different in age (*p* < 0.001) with the mean age of one being 12.50 years (range, 9–15; SD, 1.97), while the other was 17.25 years (range, 15–18; SD, 0.98).

### Biomarker identification in children and adolescents MDD

**Fig. 3:**
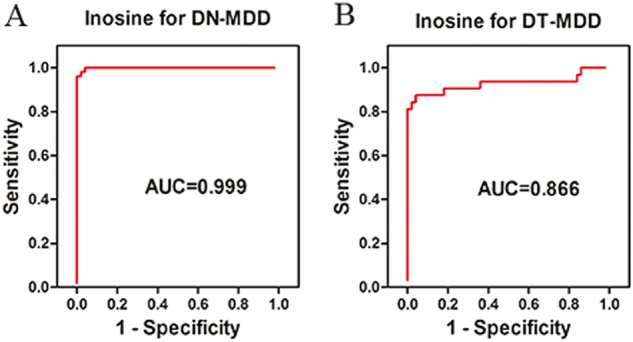
ROC curves of inosine for **a** drug-naive MDD and **b** drug-treated MDD diagnosis. AUC area under the ROC curve, MDD major depressive disorder, ROC receiver-operating characteristic

To further explore the possibility of identifying potential diagnostic biomarkers for children and adolescents with MDD, we conducted a stepwise binary logistic-regression analysis. We found that a single parameter, inosine, was significantly associated (*p* = 0.011) with children and adolescents with DN-MDD (Table S2). A decreased plasma level of inosine was correlated with an increased risk of DN-MDD in children and adolescents (odds ratio 0.01, 95% confidence interval 0.00–0.35). ROC analyses were performed to quantify the diagnostic performance of each of the 35 individual plasma metabolites previously highlighted. Inosine was identified as the most effective diagnostic biomarker for DN-MDD from HCs, with an AUC of 0.999 (Fig. 3). The ROC curves of other metabolites ranked by the AUC are presented in Figure S5. Thus, inosine was selected as an independent diagnostic biomarker for child- and adolescent-onset DN-MDD. To investigate whether inosine is affected by clinical characteristics of MDD patients, we performed additional multiple regression analysis. This identified that sex and depression symptom severity significantly influenced the plasma inosine level. These analyses suggested that plasma inosine levels are reduced more notably in boys and/or in those with more severe MDD (Table S3). Moreover, inosine was increased significantly in the DT-MDD group compared with HCs, with an AUC of 0.866 in discriminating DT-MDD from HCs (Fig. 3).

### Comparison of metabolic profiles between child and adolescent MDD and adult MDD

**Fig. 4:**
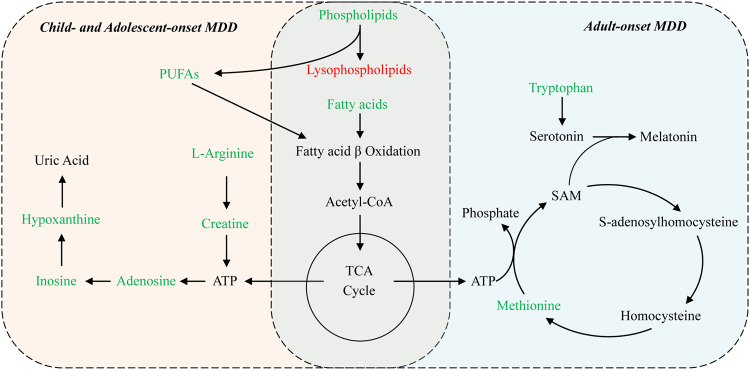
Perturbed metabolic pathways in drug-naive children and adolescents MDD and adults MDD subjects. Metabolites in red increased in the plasma, and metabolites in green decreased. ATP adenosine triphosphate, Fatty acids capric acid, cis-9-palmitoleic acid, dodecanoic acid, oleic acid, palmitic acid or stearic acid, MDD major depressive disorder, PUFAs eicosapentaenoic acid (EPA, ω-3) and arachidonic acid (AA, ω-6), SAM S-adenosylmethionine, TCA tricarboxylic acid cycle

We reanalyzed our previous metabolomics data of adult MDD [17] and compared it with the current metabolomic data from children and adolescents with MDD. The significantly perturbed pathways of both child and adolescent MDD and adults MDD are shown in Table 3. Disturbances of tryptophan and methionine metabolism were seen mainly in adults with MDD, and tryptophan was identified as an important biomarker for discriminating adult MDD. However, the child and adolescent MDD subjects exhibited significant purine metabolism disturbances, and inosine was selected as an independent diagnostic biomarker (Fig. 4).

## Discussion

To our knowledge, this is the first study to investigate potential biomarkers of children and adolescents with MDD through the use of metabolic profiling from plasma samples. In the present study, we found significant alterations in several metabolic pathways.

Levels of capric acid, cis-9-palmitoleic acid, dodecanoic acid, oleic acid and palmitic acid were decreased in children and adolescents with MDD who were drug-naive compared with healthy controls. These fatty acids are the substrates or by-products in fatty acid β oxidation pathways [24]. Carnitine (10:0), a medium-chain acyl carnitine, which supports fatty acid oxidation effectively, was also decreased. Based on these findings we suggest that downregulated fatty acid β oxidation may occur in children and adolescents with MDD. Moreover, in accordance with previous studies, both ω-3 PUFAs (EPA, ALA) andω-6 PUFAs (AA, LA) were also reduced in MDD subjects [25]. When compared to healthy controls, both EPA and inosine levels were significantly decreased in DN-MDD patients, suggesting that omega-3 PUFAs may play a role in the pathophysiology of depression by regulating the pathway of purine metabolism. This is consistent with the available scientific literature, which showed that PUFAs, including EPA and ALA, inhibit inosine 5′-monophosphate dehydrogenase activity, which is a critical purine biosynthetic enzyme [26], and then increase the subsequent catabolites (inosine and hypoxanthine) [27]. Depletion of ω-3 PUFAs have previously been reported to be associated with inflammatory processes and lipid peroxidation in the pathophysiology of depression [28]. In addition, several studies have reported that ω-3 PUFAs supplements are associated with decreased risk of depression or lower depressive symptoms in adult patients [29–31].

Metabolites involved in purine metabolism were also down-regulated in children and adolescents with MDD compared with healthy controls. These included adenosine, inosine and hypoxanthine. Uric acid, the end-product of purine metabolism, was also increased [32]. Uric acid is a major circulating antioxidant and is regarded as a compensatory mechanism to counteract oxidative stress in depression [33]. Taken together, these results suggest that purine degradation may be accelerated during depression. This may be a consequence of increased activation of xanthine oxidase and adenosine deaminase [34]. Since extracellular adenosine and its metabolite inosine, can both permeate the blood–brain barrier [35, 36], it is likely that decreased plasma levels may reflect a reduction of these metabolites in central nervous system (CNS). Adenosine and inosine modulate the release of neurotransmitters (e.g., glutamate and serotonin), synaptic plasticity and inflammatory processes through A1 and A2A adenosine receptors [37, 38]. The decreased levels of adenosine and inosine suggest that the perturbance of these physiological processes may play a role in the pathophysiology of child and adolescent depression. Moreover, creatine, an energy-related metabolite, was decreased in the present study. A previous study has also reported that creatine augmentation was associated with an improvement of depressive symptoms in adolescents with SSRI-resistant MDD [39].

We further analyzed our data to identify potential diagnostic biomarkers. Inosine was found to be the most prominent metabolite in this respect as it exhibited a significant decrease in MDD. Interestingly the discrimination based on the diagnostic performance of inosine for the DN-MDD group (AUC = 0.999) was different from that for the DT-MDD (AUC = 0.866). This was consistent with a previous study that reported that oral administration of inosine has antidepressant-like effects in animal models [40, 41]. According to our multiple regression analysis, we conclude that the plasma inosine levels are negatively correlated with depression symptoms severity, and that boys have lower inosine level than girls, which may result from the antioxidant effect of female hormones reducing the demand of uric acid in girls [42]. Therefore, as decreased levels of inosine have been previously observed in adult patients [35], low levels of inosine can be a potential diagnostic biomarker for MDD in children and adolescents. Further increased inosine after antidepressants treatment may indicate that inosine could also be a potential predictor of antidepressant effects. In future studies, more attention should be paid to inosine in children and adolescents with MDD.

The issue whether depressed children and adolescents are similar to each other is still unanswered [43]. From our findings the younger MDD patients (9–15 years) were significantly different from the older group (15–18 years). These findings may possibly question the commonly held belief that the degree of vulnerability for depression in children and adolescents changes at about 13 years of age, an age cutoff traditionally used to distinguish childhood from adolescence. Many of the neurobiological systems, such as serotonergic and adrenergic neurotransmitter systems, that are implicated in the pathophysiology of depression are not fully developed at an early age [14, 43], which may explain the differences in metabolic phenotypes between younger and elder patients. A 15-year cut-off would also be consistent with the epidemiology of adolescent depression: the prevalence dramatically increases at 15 and the sex ratio changes with more girls than boys in late adolescence [44]. We found there are several apparent differences in the metabolomic pathways associated with adult MDD [17] and child and adolescent MDD. These may help to explain the differential benefit of antidepressants, whereby antidepressants appear to be more effective for the treatment of depression in adults, than they are in children and adolescents [7, 8]. It is generally believed that the dysfunction of serotonergic system contributes to the development of depression [45], and the synthesis of serotonin in the CNS is dependent on the utilization of tryptophan—the biochemical precursor of serotonin [46]—from plasma to brain. In our study, decreased levels of tryptophan were found only in adult MDD but not in children and adolescents MDD. It is therefore possible that the differences in the clinical presentation and treatment responses in depression between young and adult patients may result at least in part from differences in tryptophan/serotonin metabolism.

There are several limitations to this study. Overall, 134 child and adolescent patients and healthy controls were recruited into this study. This relatively small sample may restrict the generalization of the findings. However, due to ethical implications related to invasive studies on children and adolescents, young patients are difficult to recruit and this is the average size of studies in the field [13–15]. Second, we found no significant differences in depression rating scale scores between drug-treated MDD and drug-naive MDD. Future studies with larger number differentiating between treatment responders and non-responders, as well as accompanying prospective studies monitoring metabolomics should be helpful. Third, despite the fact that most patients in the drug-treated MDD group were being treated with an SSRI, several other antidepressants were also used and may have contributed to heterogeneity in the metabolomics results. Fourth, no correction was made for multiple comparisons thereby increasing the potential risk for Type I errors. Fifth, there are dietary differences between China and European countries (including the much lower BMI of individuals in China), which can influence the metabolomic profiles [47, 48]. Even though all participants in this study were recruited from urban areas in China, this may affect the generalizability of the findings from this research. Finally, measurement differences relating to the use of two different depression scales based on age and the fact that biological profiling were measured at different times in the adults vs. adolescents may have contributed to errors.

In summary, we observed metabolic changes associated with several metabolic pathways, including fatty acid metabolism–particularly the PUFAs metabolism, and purine metabolism in child and adolescent MDD. We further identified inosine as a potential independent diagnostic biomarker for MDD in this population and showed that inosine levels were negatively correlated with the severity of depressive symptoms. Moreover, we found evidence to support the notion that the pathophysiology of children and adolescents with MDD was different from that of adults, especially in the metabolism of tryptophan. It is suggested that these findings contribute to improve the understanding of the pathophysiology of child and adolescent MDD and to enable improved diagnosis and treatment. Future prospective investigations with a larger number of subjects are needed to investigate their potential clinical utility of metabolic biomarkers in the diagnosis and treatment of children and adolescents with depression.

## Electronic supplementary material

Table S1(DOCX 16 kb)

Table S2(DOCX 14 kb)

Table S3(DOCX 14 kb)

Figure S1(TIF 1452 kb)

Figure S2(TIF 7046 kb)

Figure S3(TIF 4522 kb)

Figure S4(TIF 8930 kb)

Figure S5(TIF 3827 kb)

Supplemental legends(DOCX 17 kb)
