# Supplementary material for: Polyunsaturated fatty acids metabolism, purine metabolism and inosine as potential independent diagnostic biomarkers for major depressive disorder in children and adolescents
Source: Mol Psychiatry. 2018 Apr 20;24(10):1478–88. doi: 10.1038/s41380-018-0047-z (PMC6756100; doi:10.1038/s41380-018-0047-z)
Supplement: Supplementary file 1 — Table S1(DOCX 16 kb) [file 41380_2018_47_MOESM1_ESM.docx]

**Table S1.** The clinical characteristics of the younger and elder drug-naïve MDD subjects and healthy controls

|  | **Younger** | | | **Elder** | | | **P-value ^c^** |
| --- | --- | --- | --- | --- | --- | --- | --- |
|  | **DN-MDD** | **HCs** | **P-value** | **DN-MDD** | **HCs** | **P-value** |  |
| Patients (n) | 16 | 10 | / | 36 | 40 | / | / |
| Male (n, %) ^a^ | 9 (56.3%) | 8 (80%) | 0.216 | 18 (50.0%) | 19 (47.5%) | 0.828 | 0.677 |
| Age (years) | | | | | | |  |
| range | 9-15 | 7-13 | / | 15-18 | 16-18 | / | / |
| mean±sd ^b^ | 12.50±1.97 | 10.90±1.73 | 0.053 | 17.25±0.98 | 16.70±0.61 | **0.001** | **<0.001** |
| BMI (kg/m^2^) | | | | | | |  |
| mean±sd ^b^ | 19.04±2.45 | 18.40±1.47 | 0.776 | 19.87±2.17 | 20.10±1.60 | 0.346 | 0.058 |
| Depression symptoms severity | | | | | | |  |
| HAMD-17 (mean±sd, n) | NA | NA | / | 22.50±3.63 | NA | / | / |
| CDRS-R (mean±sd, n) | 45.25±5.50 | NA | / | 57.00±5.66* | NA | / | / |
| Course of illness (months) ^b^ | | | | | | |  |
| Median, IQR | 5.00 (2.50–8.00) | NA | / | 9.00 (4.50–20.83) | NA | / | 0.227 |

^a^ Analyzed by the Chi-square test.

^b^ Analyzed by Mann-Whitney U test.

^c^ Compared the younger and elder DN-MDD subjects.

* Only two elder patients were assessed using CDRS scale.

Continuous variables are expressed as mean ± standard deviation (SD) or median with interquartile range (IQR). BMI, body mass index; CDRS-R, Children’s Depression Rating Scale-Revised, DN-MDD, drug-naïve major depressive disorder; HAMD-17, Hamilton Depression Scale (17-Items); HCs, healthy controls.
