# Supplementary material for: Polyunsaturated fatty acids metabolism, purine metabolism and inosine as potential independent diagnostic biomarkers for major depressive disorder in children and adolescents
Source: Mol Psychiatry. 2018 Apr 20;24(10):1478–88. doi: 10.1038/s41380-018-0047-z (PMC6756100; doi:10.1038/s41380-018-0047-z)
Supplement: Supplementary file 2 — Table S2(DOCX 14 kb) [file 41380_2018_47_MOESM2_ESM.docx]

**Table S2.** Stepwise binary logistic-regression models for selecting potential diagnostic biomarker for children and adolescents depression patients.

| **Models** | **Parameter** | **Estimate** | **Standard Error** | **Wald Chi-Square** | **P Value** |
| --- | --- | --- | --- | --- | --- |
| **Model 1** | Intercept | 0.787 | 1.008 | 0.609 | 0.435 |
|  | Inosine | -4.622 | 1.822 | 6.436 | 0.011 |
| **Model 2** | Intercept | -5.997 | 1533.148 | 0.000 | 0.997 |
|  | Bilirubin | -28.493 | 2985.280 | 0.000 | 0.992 |
|  | Inosine | -38.618 | 2891.885 | 0.000 | 0.989 |
| **Model 3** | Intercept | -10.697 | 2174.507 | 0.000 | 0.996 |
|  | Bilirubin | -1.332 | 4965.890 | 0.000 | 1.000 |
|  | Inosine | -30.168 | 2780.874 | 0.000 | 0.991 |
|  | PC182244 | 16.239 | 4446.730 | 0.000 | 0.997 |
| **Model 4** | Intercept | -10.836 | 1942.468 | 0.000 | 0.996 |
|  | Inosine | -30.310 | 2743.343 | 0.000 | 0.991 |
|  | PC182244 | 17.322 | 2573.343 | 0.000 | 0.995 |
