# Supplementary material for: Polyunsaturated fatty acids metabolism, purine metabolism and inosine as potential independent diagnostic biomarkers for major depressive disorder in children and adolescents
Source: Mol Psychiatry. 2018 Apr 20;24(10):1478–88. doi: 10.1038/s41380-018-0047-z (PMC6756100; doi:10.1038/s41380-018-0047-z)
Supplement: Supplementary file 3 — Table S3(DOCX 14 kb) [file 41380_2018_47_MOESM3_ESM.docx]

**Table S3**. Multiple regression analysis for the clinical data of children and adolescents depression patients.

| **Variables** | **Parameter Estimate** | **Standard Error** | **t Value** | **P Value** |
| --- | --- | --- | --- | --- |
| Intercept | -1.834 | 0.865 | -2.121 | **0.039** |
| Sex | 0.446 | 0.164 | 2.719 | **0.009** |
| Age | -0.011 | 0.036 | -0.315 | 0.754 |
| BMI | 0.012 | 0.040 | 0.292 | 0.771 |
| Depression symptoms severity | -0.505 | 0.212 | -2.379 | **0.022** |
| Course of illness | 0.006 | 0.004 | 1.368 | 0.178 |

Age, body mass index (BMI) and duration of disease were regarded as continuous variables; sex (boy = 1, girl = 2) and depression symptoms severity (moderate = 1, severe = 2) were regarded as binary variables.
