# Supplementary figures and images for: Polyunsaturated fatty acids metabolism, purine metabolism and inosine as potential independent diagnostic biomarkers for major depressive disorder in children and adolescents
Source: Mol Psychiatry. 2018 Apr 20;24(10):1478–88. doi: 10.1038/s41380-018-0047-z (PMC6756100; doi:10.1038/s41380-018-0047-z)

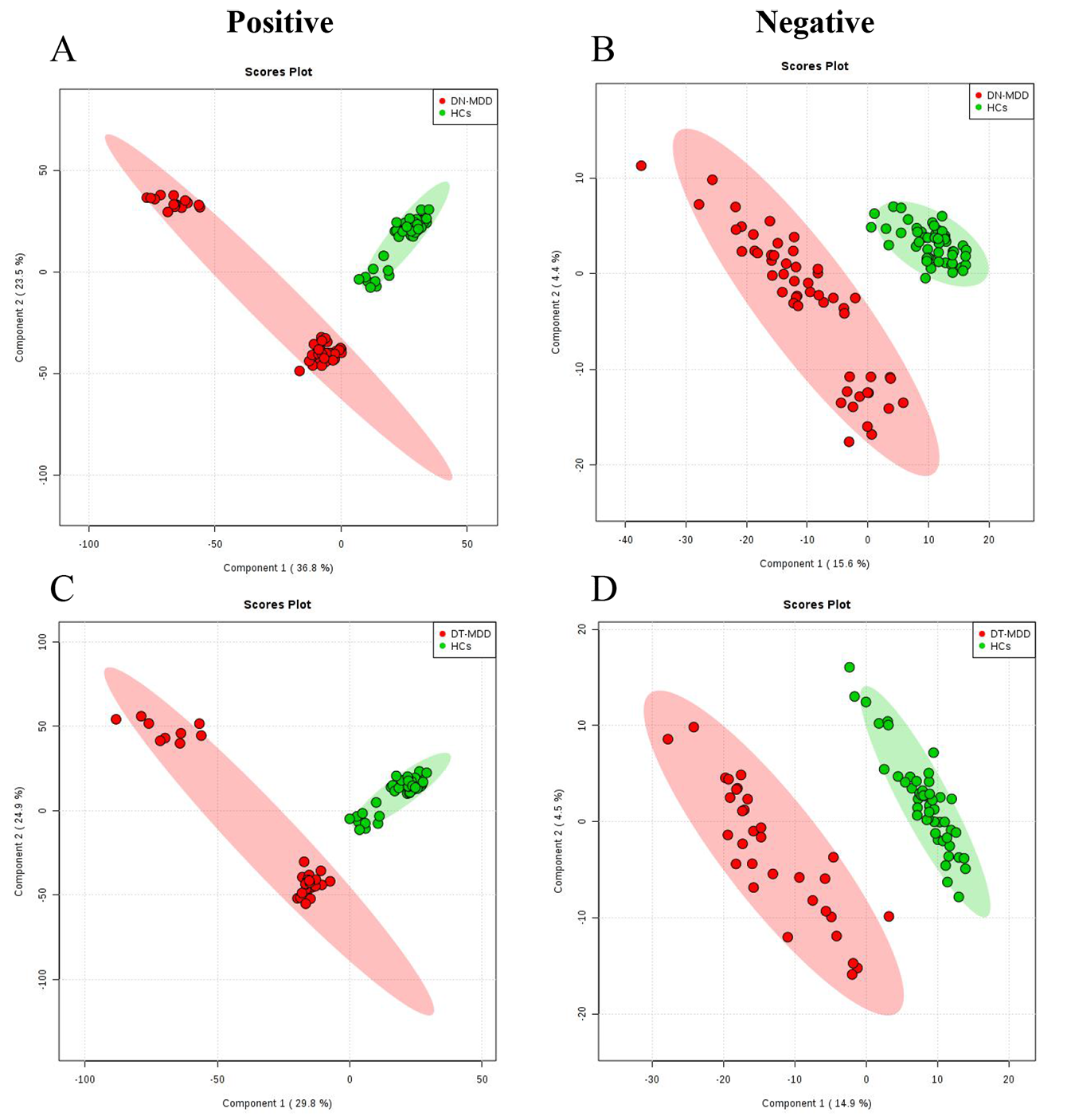

Supplement: Supplementary file 5 — Figure S2(TIF 7046 kb) [file 41380_2018_47_MOESM5_ESM.tif]

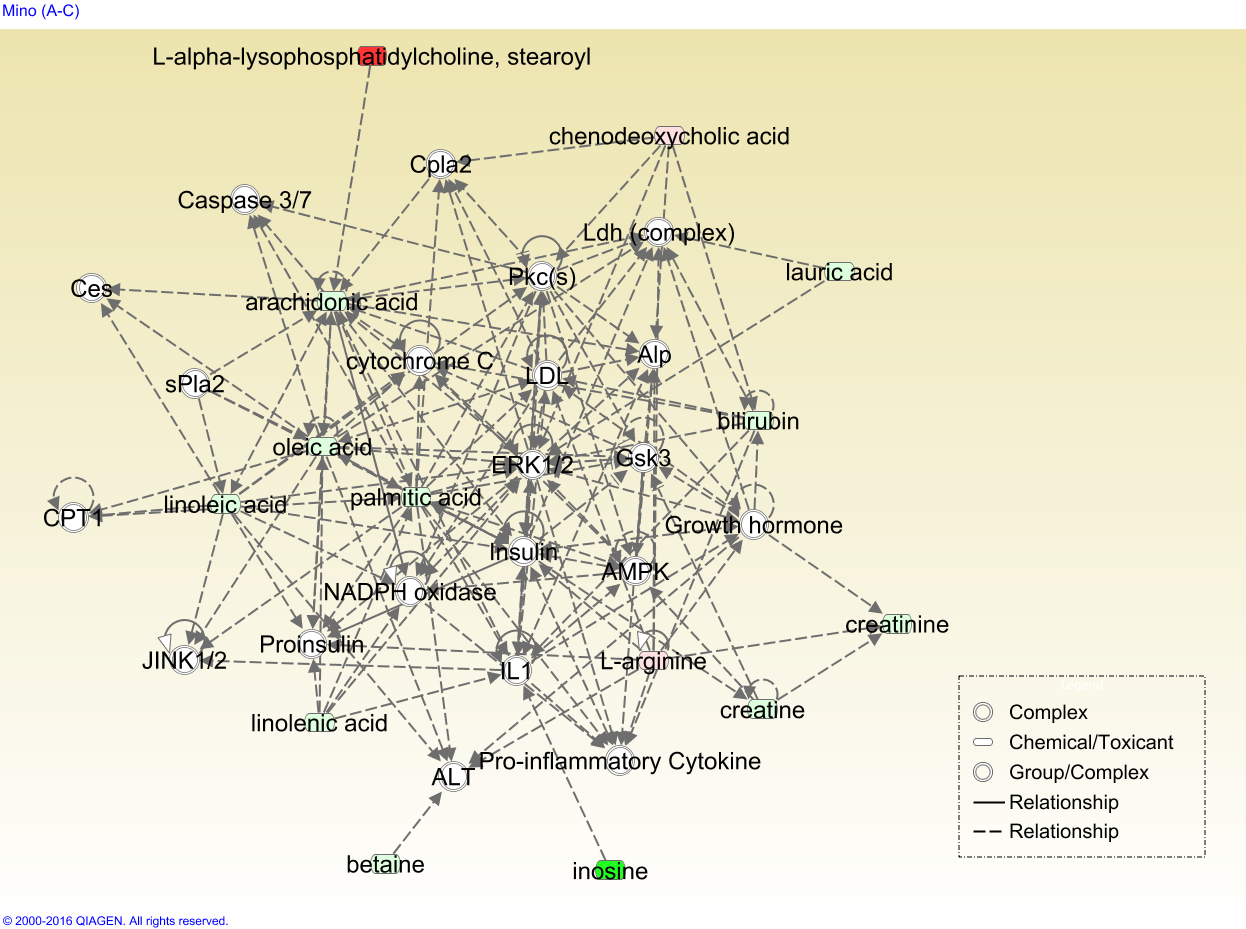

Supplement: Supplementary file 6 — Figure S3(TIF 4522 kb) [file 41380_2018_47_MOESM6_ESM.tif]

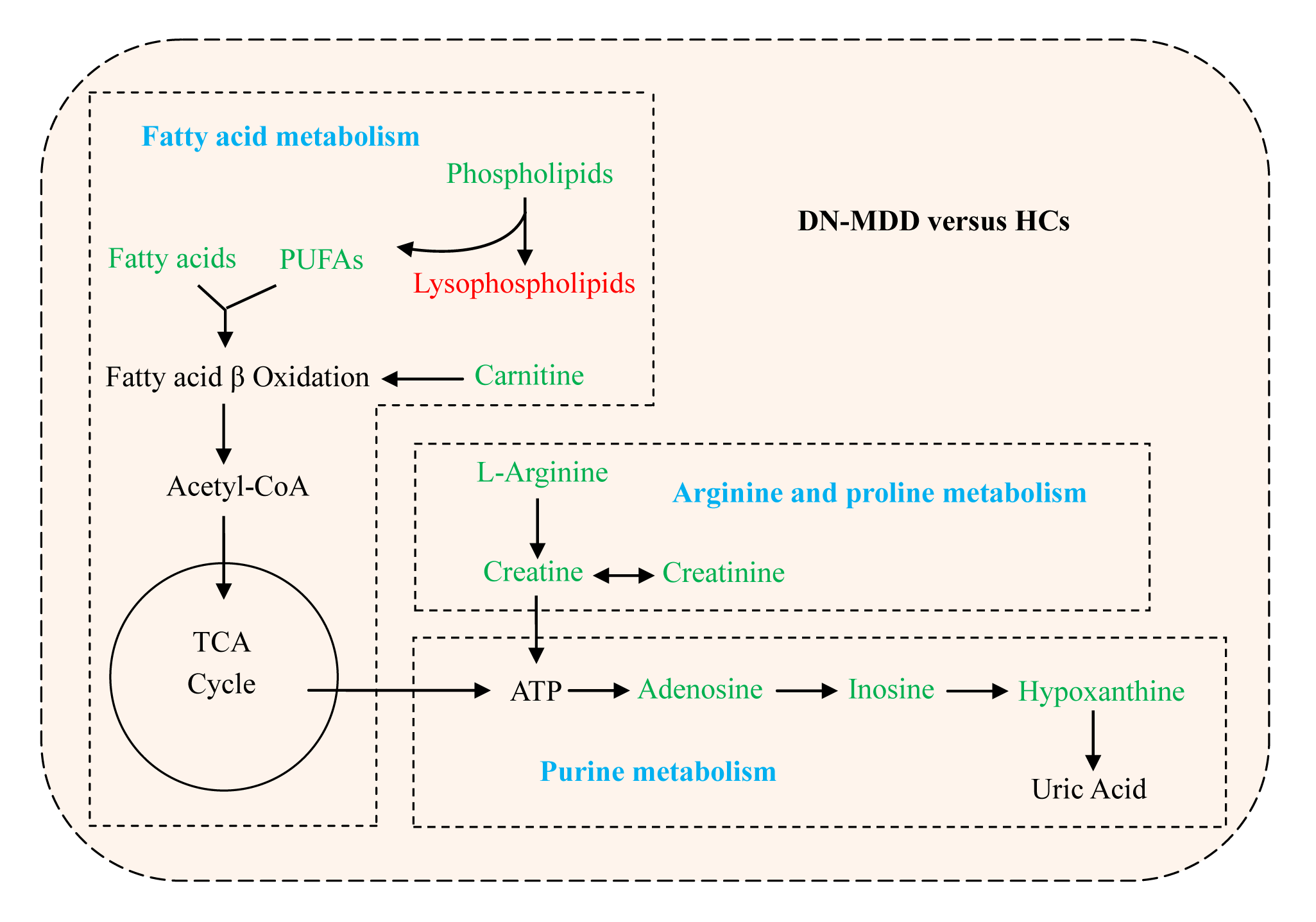

Supplement: Supplementary file 7 — Figure S4(TIF 8930 kb) [file 41380_2018_47_MOESM7_ESM.tif]
