# Supplementary material for: Polyunsaturated fatty acids metabolism, purine metabolism and inosine as potential independent diagnostic biomarkers for major depressive disorder in children and adolescents
Source: Mol Psychiatry. 2018 Apr 20;24(10):1478–88. doi: 10.1038/s41380-018-0047-z (PMC6756100; doi:10.1038/s41380-018-0047-z)
Supplement: Supplementary file 9 — Supplemental legends(DOCX 17 kb) [file 41380_2018_47_MOESM9_ESM.docx]

**Table S1.** **The clinical characteristics of the younger and elder drug-naïve major depressive disorder subjects and healthy controls.** Continuous variables are expressed as mean ± standard deviation (SD) or median with interquartile range (IQR). BMI, body mass index; CDRS-R, Children’s Depression Rating Scale-Revised; DN-MDD, drug-naïve major depressive disorder; HAMD-17, Hamilton Depression Scale 17-Item; HCs, healthy controls.

^a^ Analyzed by the Chi-square test.

^b^ Analyzed by Mann-Whitney U test.

^c^ Compared the younger and elder DN-MDD subjects.

* Only two elder patients were assessed using CDRS scale.

**Table S2. Stepwise binary logistic-regression models for selecting potential diagnostic biomarker for children and adolescents depression patients**

**Table S3**. **Multiple regression analysis for the clinical data of children and adolescents depression patients.** Age, body mass index (BMI) and duration of disease were regarded as continuous variables; sex (boy = 1, girl = 2) and depression symptoms severity (moderate = 1, severe = 2) were regarded as binary variables.

**Figure S1. Principal component analysis score scatter plots (A) in positive model and (B) in negative model.** Multivariate statistical analysis of major depressive disorder (MDD) subjects and healthy controls (HCs). DN-MDD, drug-naïve major depressive disorder; DT-MDD, drug-treated major depressive disorder; QC, quality control

**Figure S2. Partial least square discriminant analysis (PLS-DA) score plots of depression patients versus healthy controls.** PLS-DA score plots for drug-naïve major depressive disorder (DN-MDD) versus healthy controls (HCs) separation (A) in positive mode and (B) in negative mode; PLS-DA score plots for drug-treated major depressive disorder (DT-MDD) and HCs separation (C) in positive mode and (D) in negative mode.

**Figure S3.** **The most significantly altered network in drug-naïve major depressive disorder resulting from Ingenuity Pathway Analysis.** Metabolites with red symbols were increased in drug-naïve major depressive disorder compared with healthy controls, while green symbols indicate decreases. Solid lines show direct interactions between the two components, while dotted lines show indirect interactions.

**Figure S4. Perturbed metabolic pathways in first-episode drug-naïve major depressive disorder versus healthy controls.** Metabolites in red increased in the plasma, and metabolites in green decreased. ATP, adenosine triphosphate; DN-MDD, drug-naïve major depressive disorder; Fatty acids: capric acid, cis-9-palmitoleic acid, dodecanoic acid, oleic acid and palmitic acid; HCs, healthy controls; PUFAs: eicosapentaenoic acid (EPA, ω-3) and arachidonic acid (AA, ω-6); TCA, tricarboxylic acid cycle.

**Figure S5. ROC curves of differentially expressed metabolites between drug-naïve major depressive disorder versus healthy controls.** AUC, area under the ROC curve; ROC, receiver-operating characteristic.
